# Nitrogen balance and outcomes in critically ill patients: A systematic review and meta-analysis

**DOI:** 10.3389/fnut.2022.961207

**Published:** 2022-08-22

**Authors:** Yi-Bing Zhu, Yan Yao, Yuan Xu, Hui-Bin Huang

**Affiliations:** ^1^Department of Emergency, Guang'anmen Hospital, China Academy of Chinese Medical Sciences, Beijing, China; ^2^Department of Critical Care Medicine, Beijing Tsinghua Changgung Hospital, School of Clinical Medicine, Tsinghua University, Beijing, China

**Keywords:** nitrogen balance, critical illness, mortality, meta-analysis, protein

## Abstract

**Objective:**

Nitrogen balance (NB) is a commonly used nutrition indicator in clinical practice, while its relation to the interpretation of protein malnutrition and outcomes in critically ill patients remains unclear. This study aimed to evaluate the impact of NB on prognosis in such a patient population.

**Methods:**

We searched for relevant studies in PubMed, EMBASE, and the Cochrane Database up to May 10, 2022. Meta-analyses were performed to evaluate the relationship between NB (initial, final, or absolute change of NB levels) and prognosis and important clinical outcomes in critically ill patients. Pooled odds ratios (ORs) and mean differences (MDs) together with their 95% confidence intervals (CIs) were calculated. We also conducted subgroup analyses to explore the sources of heterogeneity.

**Results:**

Eight studies with 1,409 patients were eligible. These studies were moderate to high quality. When pooled, the initial NB was comparable between the survival and non-survival groups (five studies, MD 1.20, 95% CI, −0.70 to 3.11, *I*^2^ = 77%; *P* = 0.22), while a significantly higher final NB in the survival group than that in the death group (two studies, MD 3.69, 95% CI, 1.92–5.46, *I*^2^ = 55%; *P* < 0.0001). Two studies provided the absolute change of NB over time and suggested survival patients had more increased NB (MD 4.16 g/day, 95% CI, 3.70–4.61, *I*^2^ = 0%; *P* < 0.00001). Similarly, for studies utilizing multivariate logistic regression, we found an improved NB (four studies, OR 0.85, 95% CI, 0.73–0.99, *I*^2^ = 61%; *P* = 0.04) but not an initial NB (two studies, OR 0.92, 95% CI 0.78–1.08, *I*^2^ = 55%; *P* = 0.31) was significantly associated the risk of all-cause mortality. These results were further confirmed in subgroup analyses. In addition, patients with improved NB had more protein and calorie intake and a similar length of stay in hospital than those without.

**Conclusions:**

Our results suggested that an improved NB but not the initial NB level was associated with all-cause mortality in critically ill patients. This highlights the requirement for dynamic monitoring of NB during nutrition treatment. Further randomized clinical trials examining the impact of NB-guided protein intake on clinical outcomes in critically ill patients are warranted.

**Systematic review registration:**

INPLASY202250134, https://doi.org/10.37766/inplasy2022.5.0134.

## Introduction

Hypercatabolism often occurs in critically ill patients, particularly when an intense inflammatory process develops, such as severe sepsis, shock, burns, and polytrauma (1–3). Protein catabolism is a vital concomitant of critical illness and can lead to increased mortality, while severely ill survivors may have muscle weakness and physical disability lasting for years (4, 5). As the essential macronutrient, protein significantly impacts the prognosis of these patients. Some studies have suggested that protein intake, rather than other macronutrients and caloric intake, may be more relevant to clinical outcomes in critically ill patients (6).

Nitrogen balance (NB) reflects the intake or loss of whole-body protein and indicates the difference between nitrogen intake and nitrogen loss (7). It is regarded as a simple and inexpensive method and a good marker of adequate protein intake (8). Recent guidelines for nutrition therapy in critical illness recommend a high-protein diet in the intensive care unit (ICU) and the use of NB to adjust protein for each patient as a predictor of protein intake and nitrogen loss to achieve nitrogen equilibrium (9, 10).

However, despite its widespread use in clinical practice, the association between NB and the interpretation of protein malnutrition and clinical outcomes in critically ill patients remains unclear (6, 11–13). On the one hand, NB reflects only the net result of nitrogen exchange (14) and does not provide insight into the kinetics of protein synthesis, catabolism, or subtle changes in protein redistribution (12). As a result, the NB level may represent the extent of catabolism rather than the adequacy of protein intake. On the other hand, the determination of NB has its limitations from a practical point of view. NB studies require accurate measurement of protein intake and precise calculation of all sources of nitrogen excretion. However, the requirement for 24-h urine or continuous renal replacement therapy (CRRT) ultrafiltrate collection, difficulties in interpretation in acute renal injury, and even anuric and unquantified nitrogen losses (e.g., from wound drains) may confound the results in ICU patients (15). Thus, whether NB is significantly correlated with prognosis in these patients or simply as a reflection of the catabolism degree requires further clarification.

Several studies on NB in critically ill patients have been published recently (6, 16–18), though some have small or modest sample sizes. Therefore, we aimed to perform a systematic review and meta-analysis to explore the prognostic value of NB in such a patient population.

## Methods

Our study was designed based on the PRISMA checklist (Additional File 1), and the protocol has been registered on the International Platform of Registered Systematic Review (19) and Meta-analysis Protocols database (INPLASY202250134). The full text was available at https://doi.org/10.37766/inplasy2022.5.0134.

### Search strategy and selection criteria

Two independent investigators (Y-BZ and YY) systemically searched the PubMed, EMBASE, Ovid Medline, and Cochrane databases from inception through May 10, 2022, without study design and language limitations. We also searched the related websites and Google Scholar for the gray literature. Briefly, search terms included “nitrogen balance,” “nitrogen excretion,” “prognostic,” “critically ill,” “intensive care,” “survival,” and “mortality” in MeSH and keywords. The complete search strategy is attached in Additional File 2. We reviewed the citation lists of included full-text articles to avoid omitting relevant literature. Discussions between the two investigators solved disagreements.

We included studies that used NB in predicting prognosis in critically ill patients. The studies should meet the following criteria: (1) The study focused on the association between NB level (regardless of assessment timing during the study period) and the mortality risk in adult (≥18 years old) patients; (2) The outcome data included any reporting form of survival data [i.e., pooled odds ratios (ORs) or hazard ratios (HRs) or mean differences (MDs)] that could be extracted; and (3) The study design was limited to cohort, case-control, or randomized clinical trials (RCTs). We excluded studies that did not report clear NB definitions, provided without prognostic outcomes, and focused on animal experiments, children, or pregnant women. Studies available only in review, abstract, meeting reports, or comments were also excluded. Notably, we checked the specific study period and inclusion criteria for the potentially relevant studies from the same group research team to ensure that these are all unique cohorts with no overlap.

### Outcomes

The primary outcome in the present meta-analysis was all-cause mortality at the longest follow-up available. We defined the survival patients as those still alive at the end of follow-up, as opposed to non-survival patients. The secondary outcomes included calorie and protein intake during the study period and length of stay (LOS) in the ICU and hospital.

### Data extraction and quality assessment

Y-BZ and YY independently collected the following data from the original studies: study characteristics (name of the first author, study design, NB definition, country published year, etc.), patient characteristics (sample size, age, patient source, BMI, sex distribution, disease severity, etc.) and outcomes assessment (any survival data, methodological quality).

The above two investigators evaluated the quality of each included study using the Newcastle-Ottawa Scale (NOS) for cohort studies (20). High, moderate, and low quality was defined if studies were classified as scores of 8–9, 6–7, and <6, respectively. Discrepancies were identified and resolved through discussion.

### Statistical analysis

We combined the results from all relevant studies to investigate the pooled odds ratio (OR) and associated 95% confidence intervals (CIs) for dichotomous outcomes (i.e., all-cause mortality) and to estimate the mean differences (MD) and 95% CI as the effective results for continuous outcomes (i.e., NB levels, ICU or hospital LOS, and protein or calorie intake). Before data analysis, we estimated the mean from the median and standard deviations (SD) from IQR using the previous study's methods, if required (21).

In the primary analysis, we separately conducted two types of meta-analyses according to the different reporting forms of NB risk in the included studies. (1) The strength of association of NB [initial, final, or change of NB over time (defined as absolute changes between initial and final NB)] with the mortality risks was measured by differences in means (difference in means/pooled SD) between the survival and non-survival groups. (2) As to studies utilizing regression analyses to investigate the relationship between NB and mortality, we combine the mortality estimates with corresponding standard errors by the generic inverse variance method. Thus, these studies' ORs required natural logarithmic transformations before merging. When both multivariate and univariate results were available, the former was preferred in our analysis.

We tested the heterogeneity across included studies using the *I*^2^ statistic. An *I*^2^ ≥ 50% indicates significant heterogeneity, and a random-effect model was used, whereas a fixed-effect model for *I*^2^ < 50%. Publication bias was performed when at least ten studies were included in the meta-analysis (22). In all analyses, we used RevMan version 5.4 (Cochrane Collaboration, Oxford, UK).

### Additional analyses

To explore the potential influence factors for the primary outcome, we performed subgroup analyses by pooling studies with the following properties: (1) geographic location: Asian or not Asian countries; (2) sample size: >100 or ≤100 (23); (3) acute kidney injury (AKI) percentage: ≥50 or <50%; (4) CRRT percentage: ≥50 or <50%; (5) study design: prospective or retrospective study; (6) mortality prevalence≥40 or <40%; and (7) study quality (NOS): >7 or ≤7. We defined the cut-off values of AKI%, CRRT%, and mortality% based on the approximate average of these variables. Subgroup analyses were conducted when more than three studies were available.

## Result

### Study selection

**Figure 1:**
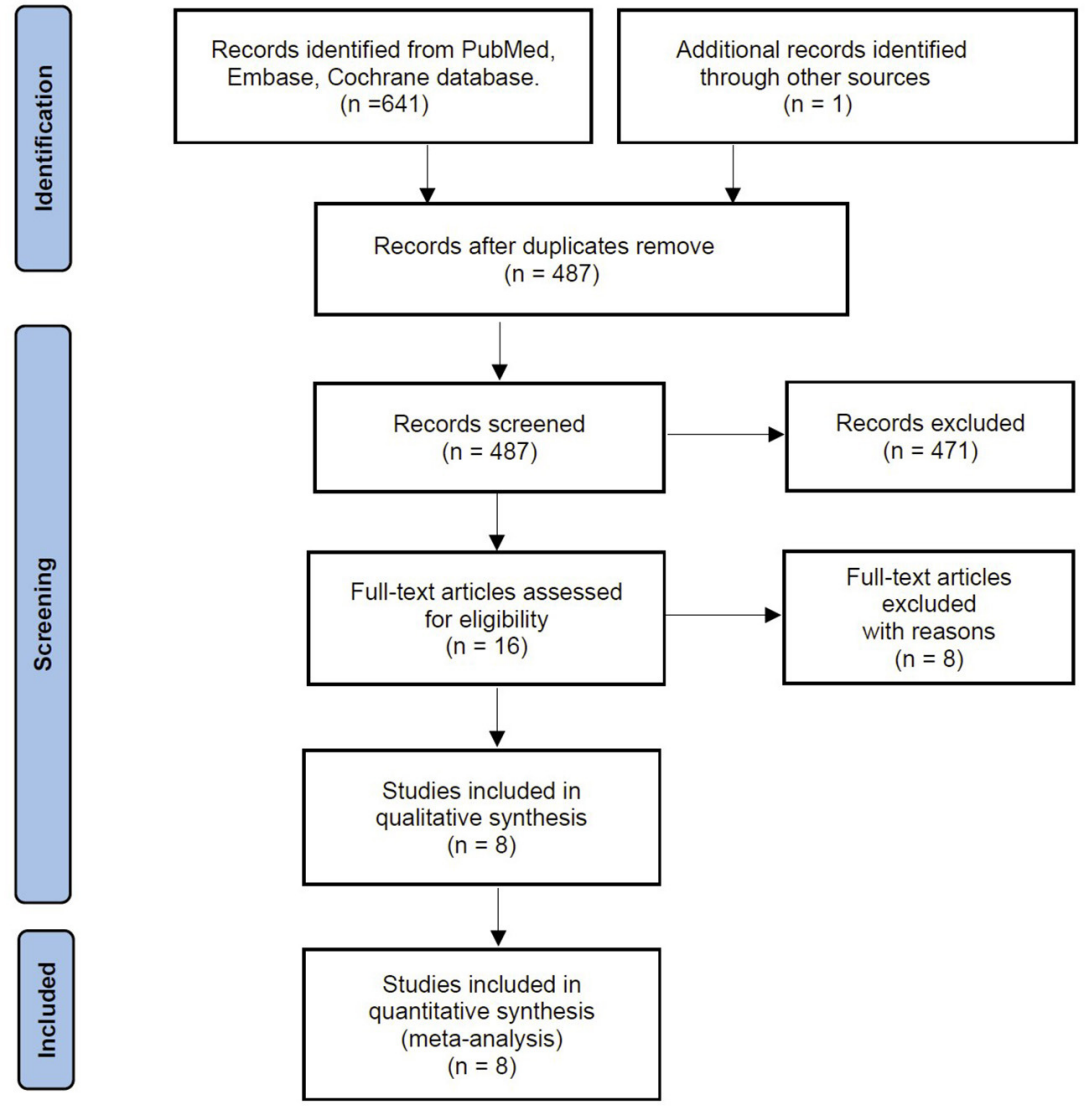
Flow chart of literature selection.

Our search identified 641 citations from predefined databases. After screening the titles and abstracts, 16 were qualified for full-text review. Based on the full-text evaluation, we excluded eight studies for exclusion reasons based on the full-text review (Appendix File 3). Thus, the remaining eight studies with 1,409 patients were included in the final meta-analysis (6, 13, 16–18, 24–26) (Figure 1).

### Study characteristics and methodological quality

Table 1 presents the characteristics of the eligible studies. All these studies were observational and were published between 2003 and 2021 in five countries [Brazil n = 4 (17, 24–26), Thailand *n* = 1 (6), Korea *n* = 1 (18), USA *n* = 1 (16), and Australia *n* = 1 (13)]. Five studies focused on ICU patients (6, 13, 16–18), and the other three recruited more than 60% of patients requiring ICU admission (24–26). Most patients had AKI (71.7%, 1,011/1,409) (6, 13, 17, 24–26), and especially, three studies recruited only patients requiring CRRT (6, 13, 26). The follow-up period varied among the studies, with five selected hospital stays (13, 16, 18, 24, 25), while the other three selected ICU stays (17), 28-day (6), and 30-day (26), respectively. The included studies have a mean mortality rate of 43% (ranging from 17.7 to 68.2%). As to NB assessment, six studies provided NB levels between survival and non-survival patients (6, 13, 17, 24–26), while two studies compared the mortality rates between patients with or without positive NB (16, 18). Five studies used regression analyses to investigate the relationship between NB and mortality (6, 13, 18, 24, 26).

**Table 1:** Characteristics of included studies in the current meta-analysis and systemic review.

**References**	**Country**	**Design**	**Pts**	**ICU AD (%)**	**AKI (%)**	**RRT (%)**	**Sample size**	**Mortality (%)**	**Age (years)**	**Male (%)**	Initial NB^*^(g/day)
Scheinkestel et al. (13)	Australia	P, SC	Ventilated pts requiring CRRT	100	100	100	50	26.0	53.3	31.0	−1.35
Ponce et al. (26)	Brazil	P, SC	Pts with HVPD	70	100	100	130	66.5	54.1	71.5	−7.13
Berbel et al. (24)	Brazil	P, SC	Pts with AKI	66.9	100	0	133	26.3	61.1	70.9	−5.41
Kritmetapak et al. (6)	Thailand	P, SC	Pts with AKI+CRRT	100	100	100	70	61.4	60.7	67.1	−10.8
Felicetti-Lordani et al. (17)	Brazil	P, MC	General ICU pts	100	17.9	0	234	23.9	52.5	63.7	−11.52
Bufarah et al. (25)	Brazil	P, SC	Pts with AKI	62.1	100	52	595	46.1	64.0	64.5	−3.38
Kim et al. (18)	Korea	R, SC	Pts in NICU	100	0	0	175	17.7	50.8	50.3	−7.21
Buckley et al. (16)	USA	R, SC	COVID-19	100	0	0	22	68.2	66.0	64.0	−12.1

* Defined as the mean or median of all patients in the included study.

AD, admission; AKI, acute kidney injury; COVID-19, Corona Virus Disease 2019; HVPD, high-volume peritoneal dialysis, ICU, intensive care unit; MC, multi-centers; NB, nitrogen balance; P, prospective; pts, patients; R, respective; CRRT, continuous renal replacement therapy; SC, single center.

The details of the quality assessment are available in Additional File 4, with the study quality ranging from moderate to high (scores ranging from 7 to 9). Overall, seven studies were classified as high quality and the other one as moderate quality. We did not evaluate the publication bias for less than ten studies included.

### Primary outcome

**Figure 2:**
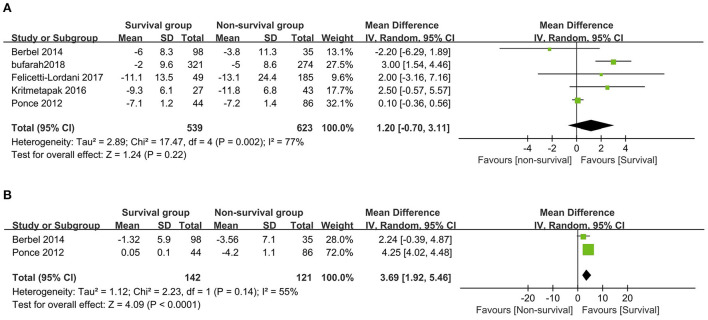
The forest plot in assessing the initial nitrogen balance **(A)** and the final nitrogen balance **(B)** levels between survival and non-survival patients. CI, confidence interval; IV, inverse variance; SD, standard deviation.

A total of six studies compared NB levels between survival and non-survival patients (6, 13, 17, 24–26), of which five provided data of initial NB or final NB that could be pooled (6, 17, 24–26). When pooling these studies, we found that the initial NB was comparable between the survival and non-survival groups (five studies, *n* = 1,162; MD 1.20 g/day, 95% CI, −0.70 to 3.11; *I*^2^ = 77%, *P* = 0.22) (Figure 2A), while a significantly higher final NB in survival group patients than that in non-survival group patients (two studies, *n* = 263; MD 3.69 g/day, 95% CI, 1.92–5.46; *I*^2^ = 55%, *P* < 0.0001) (Figure 2B). The remaining study by Scheinkestel et al. recruited 50 consecutive critically ill patients requiring CRRT and reported that patients achieving a mean positive NB (0.2 g/d) during the study period had significantly improved survival than those without (−4.1 g/d) (13). Subsequently, we conducted subgroup analyses to explore potential confounding factors. In terms of between-groups analyses, initial NB was not associated with a higher risk of mortality in all the subgroups, including geographic location, AKI percentage, CRRT percentage, sample size, study design, or mortality prevalence (all *P* values ranging from 0.15 to 0.84 with *I*^2^ ranging from 21 to 87% (Table 2).

**Table 2:** Summary of the nitrogen balance and Nutrition data in the included studies.

**References**	**Group**	**Nitrogen balance, gN/day**	**Intake protein, g/kg/day**	**Intake calorie, kcal/kg/day**
Scheinkestel et al. (13)	S/NS:13/37	T: 0.04/4.8	Control group: 2.0 for 6 days Trial group: 1.5 for 2 d, 2.0 for 2 d, and then 2.5 for the final 2 d.	According to the Schofield equation and energy expenditure measured by metabolic cart if available
Ponce et al. (26)	S/NS: 44/86	I: −7.1 ± 1.2/−7.2 ± 1.4 F: 0.05 ± 0.1/−4.2 ± 1.1	Dietary protein was calculated from a 24-h dietary intake of patients closely supervised by a renal dietician.	NA
Berbel et al. (24)	S/NS: 98/35	I: −6 (−11.1, 0.16)/−3.76 (−14.8, 0.42) F: −1.32 (−2.84 to 5.10)/−3.56 (−6.96, 2.56)	I: 0.54 (0.24–0.99)/0.3 (0.09–0.628) F: 1.13 (0.78–1.4)/1.15 (0.59–1.48)	I: 12.9 (5.6, 22.3)/7.2 (2.15, 14.8) F: 23.9 (18, 29.9)/25.6 (9.39, 28.6)
Kritmetapak et al. (6)	S/NS: 27/43	I: −9.3 ± 6.1/−11.8 ± 6.8	I: 0.8 ± 0.2/0.5 ± 0.3	NA
Felicetti-Lordani et al. (17)	S/NS: 185/49	I: −11.1 ± 13.5/−13.1 ± 13.4	Majority of the patients had protein loss between 0 and −5 g (41.0%) or between −5 and −10 g (23.1%); 17.5% had severe hypercatabolism.	Defined by the assistant team
Bufarah et al. (25)	S/NS: 321/274	I: −2 (−9, 4)/ −5 (−12, −0.4)	I: 0.8 (0.4, 1.2)/0.4 (0.05, 0.9)	I: 17.1 (8.2, 24.2)/8.5 (1.4, 19.8)
Kim et al. (18)	INB/NINB: 39/38	I: −9.3 ± 6.5/−8.9 ± 6.5	I: 0.66 ± 0.56/0.54 ± 0.48 T: 1.94 ± 0.63/1.28 ± 0.54	I: 12.0 ± 10.1/10.4 ± 9.0 T: 25.3 ± 7.5/21.5 ± 7.9
	PNB/NPNB: 35/140	I: 3.2 ± 3.8/−9.8 ± 6.3	T: 1.58 ± 0.4/0.58 ± 0.48	T: 25.6 ± 7.3/11.7 ± 9.5
Buckley et al. (16)	ANB/NNB: 5/24	T: 5.3 ± 7/−15.7 ± 7.6	T: 1.2 ± 0.4/0.8 ± 0.8	T: 17 ± 3/11 ± 9

ANB, Achieved nitrogen equilibrium; F, final evaluation; I, initial evaluation; INB, improved nitrogen balance; NANB, NINB, Nonimproved nitrogen balance; NNB, Negative nitrogen balance; PNB, positive nitrogen balance; S, survival patients; NS, non-survival patients; T, During the total follow-up.

**Figure 3:**
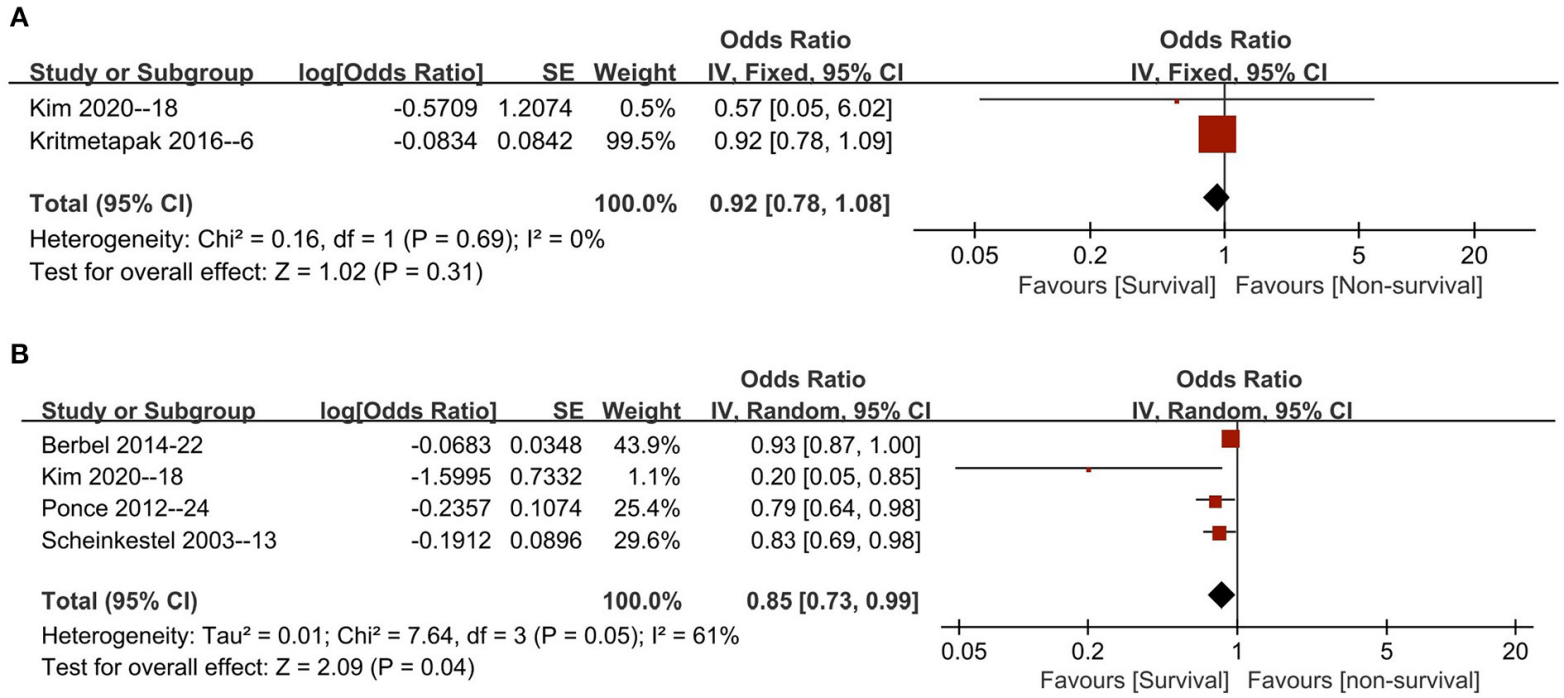
The pooled estimate of OR of all-cause mortality with initial nitrogen balance **(A)** and final nitrogen balance **(B)** in critically ill patients. CI, confidence interval; IV, inverse variance; SD, standard deviation.

Five studies reported ORs utilizing multivariate logistic regression for the risk estimation of NB in patients (6, 13, 18, 24, 26). All these studies provided ORs based on initial NB or improved NB levels that could be pooled. The pooled results suggested that initial NB values were not associated with mortality [two studies (6, 18), *n* = 245; OR 0.92, 95% CI 0.78–1.08, *I*^2^ = 0, *P* = 0.31] (Figure 3A), while an improved NB significantly decreased mortality (four studies, *n* = 488; OR 0.85, 95% CI, 0.73–0.99; *I*^2^ = 61%, *P* = 0.04) (13, 18, 24, 26) (Figure 3B). Similarly, in the subgroup analyses, the improved NB measurement was associated with significantly increased survival when pooled studies focused on patients based on most of the predefined subgroups (all *P* values ranging from 0.002 to 0.03 with *I*^2^ ranging from 39 to 77%) except in subgroups of RRT percentage ≥50% (*p* = 0.38) and mortality prevalence ≥40% (*p* = 0.15) (Table 3).

**Table 3:** Subgroup analyses of the primary outcome in the current meta-analysis.

		**References**	**Number of patients**	**MD/OR (95 % CI)**	**Test for subgroup differences**	***I*^2^, %**	***P***
**Subgroups of the between group initial nitrogen balance**							
Geographic location	Asian	(6)	70	2.50 (−0.57, 5.57)	Chi^2^ = 0.68, *P* = 0.41, *I*^2^ = 0%	–	0.11
	Non-Asian	(17, 24–26)	1,092	0.91 (−1.27, 3.10)		81	0.41
Sample size	>100	(6)	70	2.50 (−0.57, 5.57)	Chi^2^ = 0.68, *P* = 0.41, *I*^2^ = 0%	–	0.11
	≤ 100	(17, 24–26)	1,092	0.91 (−1.27, 3.10)		81	0.41
Study design	Prospective	(6, 17, 24–26)	1,162	1.20 (−0.70, 3.11)	Not applicable	77	0.22
	Retrospective	–	–	–		–	–
AKI percentage	<50%	(17)	234	2.00 (−3.16, 7.16)	Chi^2^ = 0.10, *P* = 0.75, *I*^2^ = 0%	–	0.45
	≥50%	(6, 24–26)	928	1.11 (−0.398, 3.20)		82	0.30
RRT percentage	<50%	(17, 24)	367	−0.41 (−4.48, 3.66)	Chi^2^ = 0.78, *P* = 0.38, *I*^2^ = 0%	36	0.84
	≥50%	(6, 25, 26)	795	1.70 (−0.62, 4.02)		87	0.15
Mortality prevalence	<40%	(17, 24)	367	−0.41 (−4.48, 3.66)	Chi^2^ = 0.78, *P* = 0.38, *I*^2^ = 0%	36	0.84
	≥40%	(6, 25, 26)	795	1.70 (−0.62, 4.02)		87	0.15
Study quality	>7	(6, 17, 24–26)	1,162	1.20 (−0.70, 3.11)	Not applicable	77	0.22
	≤ 7	–	–	–		–	–
**Subgroups of the regression analyses of nitrogen balance and mortality**							
Geographic location	Asian	(18)	175	0.20 (0.05, 0.85)	Chi^2^ = 3.99, *P* = 0.05, *I*^2^ = 75%	–	0.03
	Non-Asian	(13, 24, 26)	313	0.88 (0.79, 0.98)		42	0.02
Sample size	>100	–	–	–	Not applicable	–	–
	≤ 100	(13, 18, 24, 26)	488	0.91 (0.86, 0.96)		61	0.0008
Study design	Prospective	(13, 24, 26)	313	0.88 (0.79, 0.98)	Chi^2^ = 3.99, *P* = 0.05, *I*^2^ = 75%	42	0.02
	Retrospective	(18)	175	0.20 (0.05, 0.85)		–	0.03
AKI percentage	<50%	(18)	175	0.20 (0.05, 0.85)	Chi^2^ = 3.99, *P* = 0.05, *I*^2^ = 75%	–	0.03
	≥50%	(13, 24, 26)	313	0.88 (0.79, 0.98)		42	0.02
RRT percentage	≥50%	(13, 26)	313	0.52 (0.12, 2.23)	Chi^2^ = 0.36, *P* = 0.55, *I*^2^ = 0%	77	0.38
	<50%	(18, 24)	175	0.81 (0.71, 0.93)		0	0.002
Mortality prevalence	<40%	(24)	50	0.79 (0.64, 0.98)	Chi^2^ = 0.34, *P* = 0.56, *I*^2^ = 0%	–	0.03
	≥40%	(13, 18, 24)	438	0.86 (0.70, 1.06)		66	0.15
Study quality	>7	(13, 18, 24, 26)	488	0.85 (0.73, 0.99)	Not applicable	61	0.04
	≤ 7	–	–	–		–	–

AKI, acute kidney injury; CI, confidence interval; MD, mean difference; NA, not applicable; RRT, renal replacement therapy.

Two studies provided the change of NB over time (absolute changes between initial and final NB, defined by the authors) between the survival and non-survival groups (24, 26). The pooled result suggested survival patients had more NB increased (2 studies, *n* = 263; MD 4.16 g/day, 95% CI, 3.70–4.61; *I*^2^ = 0%, *P* < 0.00001) (Additional File 5).

In addition, Kim et al. recruited 175 neurocritically ill patients and suggested that the positive NB (NB ≥ 0 g/day) group had fewer events of in-hospital mortality (5.7 vs. 20.7%; *P* = 0.038) and fewer neurological worsening (5.7 vs. 24.3%; *P* = 0.015) than those in the negative NB group, while one small included study (*n* = 22) showed that hospital mortality was comparable between patients with or without achieving nitrogen equilibrium (−4 to +4 g/day) (80 vs. 65%, *P* = 1.0).

### Secondary outcomes

Three studies investigated the relationship between the NB and protein intake, of which the study by Scheinkestel et al. (13) suggested that adjusted NB level was positively related to protein intake (*P* = 0.0075). The other two studies provided specific data on this topic and pooled the results showed that patients with improved NB levels had more protein intake than those without achieving improved NB (MD 0.60 g/kg/day, 95% CI, 0.37–0.83; *I*^2^ = 0%, *P* < 0.00001) (16, 18) (Additional File 6). Two studies found that patients with an improved NB had more calorie intake (MD 4.62 kcal/kg/day, 95% CI, 1.90–7.35; *I*^2^ = 0%, *P* = 0.0009) (Additional File 7), while a similar hospital LOS (MD 6.51 days, 95% CI, −7.84 to 20.87; *I*^2^ = 0%, *P* = 0.37) (Additional File 8) when compared with those without improved NB (16, 18). Only one study reported no difference in ICU LOS between patients with or without an improved NB after treatment (18.0 vs. 20.5 days, *P* = 0.815) (18).

## Discussion

The current meta-analysis evaluated the association of NB level with the mortality risk of critically ill patients based on eight published studies. We found that many patients presented with negative nitrogen or protein balance. Our pooled results demonstrated that achieving an improved NB, but not an initial NB level, has predictive prognosis value in critically ill patients. Further subgroup analyses confirmed these findings. Moreover, patients with improved NB levels were administered higher protein and calories during the study period than those without improvement in NB. No difference was found between the two groups in hospital LOS.

### Explain the results of our research

Our study found that finial NB rather than initial NB was associated with mortality, which can be explained by stress metabolism for adaptive response to acute disease and the timing of protein intake during the treatment period. In the initial stages of severe illness, protein metabolism alters into catabolism. Negative NB at this stage is common, as shown by a mean initial NB of −7.2 g N/day from all included studies (Table 1). Particularly, in their cohort of 234 adults, Felicetti-Lordani et al. found no NB-positive patients when assessing the first NB within 24 h of ICU admission (17). The decomposition and liberation of skeletal muscle proteins result in a sustained loss of muscle tissue accompanied by an increase in the synthesis of numerous acute-phase proteins (6, 24, 25). It is demonstrated that among the critically ill patients, muscle wasting occurred early and rapidly during the first week of critical illness (nearly 20% reductions in the rectus femoris cross-sectional area observed after 10 days of ICU admission), depending on the intensity of catabolism and the number of organ failure (27).

However, it should be noted that the early increased supply of amino acids cannot interrupt the catabolic phase and may even exacerbate adverse consequences in these patients. Recent reports in the literature have proposed the concept of autophagy to provide a physiological explanation for the poor prognosis of early high protein intake (28–30). Under the stress phase, the cells selectively eliminate damaged organelles or proteins *via* autophagy to maximize responses to oxidative stress, maintain cell structure, promote protein synthesis, and improve outcomes (30). Thus, an excessive supply of amino acids, as potent autophagy inhibitors at this stage, will undoubtedly exacerbate damage to the cells (31) and lead to poor clinical prognosis (32, 33). However, a recent systematic review and meta-analysis that included 19 RCTs found that higher protein delivery (1.3 ± 0.48 g/kg/day) did not significantly affect overall mortality compared with lower protein delivery (0.9 ± 0.3 g/kg/day) in critically ill patients (19.8 vs. 22.2%, *P* = 0.34) (34). Of note, 16 of the 19 included RCTs initiated protein delivery within 3 days of ICU admission, without evidence of deleterious effects. In contrast, the higher protein was associated with a trend toward a shorter MV and ICU LOS duration. In addition, Pooled results of five small studies suggested that higher protein delivery was associated with a significant reduction in muscle loss of 3.4% per week. Therefore, early enteral nutrition should not be interfered with or delayed by maintaining autophagy in the first week of ICU admission (28, 35).

By contrast, it has been widely accepted that more amino acids are required to provide substrates for protein synthesis in the relatively stable and later stages of severe illness. Moreover, critically ill patients have a higher threshold for anabolism, so more protein needs to be provided to match their synthesis rate to promote positive NB, reduce inflammation and organ damage, and improve immune function. Our results revealed an improved NB was associated with higher protein intake and significantly improved survival. Thus, our study provides evidence for these theoretical and clinical studies. These mechanisms based on continuous monitoring of NB levels and optimal protein supply may be associated with improved outcomes in critically ill patients.

### Current literature and future research

Several aspects of using NB in critically ill patients are worth discussing. First, we found a considerable variation in the initial NB among the included patients (6, 13, 16–18, 24–26). For CRRT patients, higher negative NB values prevailed (−7.13 to −10.8 g/day) (6, 26), which, in addition to excessive catabolism, are associated with the loss of amino acids during the treatment of CRRT. In contrast, for patients without CRRT, the levels of NB are more complex. Buckley et al. found significantly higher negative values (−12.1 g/day) in critically ill ventilator-dependent patients with COVID-19 (16). In contrast, Felicetti-Lordani, in their inclusion of 234 ICU patients, found that patient type and reason for ICU admission were strongly associated with NB levels (17). Negative NB values were higher in trauma and medical patients and less negative in elective postoperative surgery patients. Interestingly, the authors also found that APACHE II was not associated with NB values in this cohort of patients (17). Although only from a few cohorts, these results suggest the need for individualized monitoring of NB in critically ill patients.

Second, emphasis should be placed on protein intake, particularly in CRRT patients. Current ESPEN guidelines recommend providing protein at a dose of 1.5 g/kg/day in critically ill patients with severe metabolic processes (9). When CRRT is used, the dose should be increased by 0.2 g/kg/day to a maximum of 2.5 g/kg/day (36). However, four included prospective studies enrolled CRRT patients (6, 13, 25, 26), three of which did not prescribe protein intake based on NB monitoring results (6, 25, 26). This resulted in extremely low protein intakes in the recruited patients. Notably, two of these studies showed protein intakes of only 0.62 and 0.61 g/day, and these studies ultimately failed to achieve positive NB (−2.76 and −2, respectively) (6, 25). In contrast, in the Scheinkestel study, patients received three continuous protein feeding regimens of 1.5, 2.0, and 2.5 g/kg/d (13). This cohort had a better positive NB (nearly 50% of measurements). Notably, the mortality rate in the three low protein intake studies was 58% (46.1–66.5%) (6, 25, 26), much higher than that in the study by Scheinkestel et al. (26.3%) (13).

Third, it is worth exploring how increased protein intake can improve NB and thus provide clinical benefits to critically ill patients. Several new high-protein formulations are now available to help meet nutritional goals (37, 38). These formulas help patients reach their protein intake goals faster, with higher serum amino acid concentrations and good intestinal tolerability and safety (39, 40). Some studies suggest that “special formulas” are needed for patients with severe diseases, partly based on the different amino acid content of acute-phase proteins compared to structural or transit proteins (41). Therefore, infusion of a regular amino acid formula diet may not be sufficient to meet the higher requirements for certain essential amino acids. Conversely, an excess of other amino acids can cause a metabolic burden because there is no pool of amino acid stores in the body.

On the other hand, achieving protein goals should avoid overfeeding. A recent prospective observational study by Singer's team demonstrated the feasibility of attaining protein intake goals guided by 24-h urinary nitrogen excretion in a highly catabolic population and achieving a more stable NB (*P* = 0.03); while administering enteral nutrition formulas with high protein-to-energy ratios to avoid overfeeding (42).

In recent years, it has been noted that the therapeutic effects of high protein may be more pronounced under exercise conditions (43). Trials combining high protein intake with exercise have shown that better muscle strength or function can be maintained than nutrition or exercise alone in healthy adult subjects (43) or patients recovering from critical illness (44). A recent RCT that included 181 critically ill patients found that high protein intake combined with resistance exercise produced higher protein intake (*P* < 0.0001), higher body composition summary scores at 3 months (*P* = 0.01), and 6 months (*P* = 0.01), and reduced mortality (45). Another larger RCT study is underway (46).

Finally, only one RCT, the EAT-ICU Study (43), investigated the effect of a combined energy-protein nutrition regimen based on energy goals determined by indirect calorimetry and daily NB. However, the authors found no differences in the physical component summary score of SF-36 and other clinical outcomes at 6 months between early goal-directed nutrition and standard of care patients. The absence of benefits in this RCT can be explained by including a high proportion (47%) of septic patients, excluding more malnourished patients, and insufficient statistical power for their clinical outcomes (47). Therefore, more RCTs with large sample size, reasonable energy-protein targets, and clear target populations are needed in the future to evaluate the impact of NB-guided protein intake on clinical outcomes.

### Strengths and limitations

To the best of our knowledge, this study is the first systematic review and meta-analysis investigating the impact of NB level on clinical outcomes. Our study supports previous guidelines; that is, dynamic assessment of NB is required, and protein intake can be adjusted based on NB in critically ill patients. However, several limitations must be considered in our meta-analysis. (1) The observational design of all included studies excluded any causal inference. Meanwhile, only patients who underwent NB testing were included in retrospective studies, prone to selection bias. (2) Some studies assessed either initial NB or final NB levels, ignoring assessments of NB levels over time (6, 13, 23). Moreover, the patient type and the ICU admission cause might correlate with NB levels in the included studies (17). (3) Some studies lack a detailed description of Nb measurement methods and results. (4) Three studies included a certain proportion of non-ICU patients (30–37%) (22–24). However, 95% (819/858) of these patients had AKI, while 84% (725/858) received CRRT. The three cohorts had a mean mortality rate of more than 45%. (5) A large proportion of included studies recruited patients with AKI in our meta-analysis, potentially affecting the representative for general ICU patients. In the subgroup of the regression analyses, we found that studies with an RRT% <50% were significantly related to mortality than RRT% ≥50%. (5) No research has examined whether altering protein consumption to enhance NB would impact outcomes, and most studies did not randomly assign patients to receive a certain protein intake. (6) In the subgroup analyses, we could not have considered all the confounding factors that might play a role in linking NB levels to all-cause mortality, such as the effects of disease severity, calorie intake, nutritional status, and feeding approach. Finally, only a few studies were included in the subgroup analyses, which may lead to deviations in the results.

## Conclusions

In conclusion, the current study suggested that improved NB was associated with a better prognosis in critically ill patients. Whether nutritional support can reverse catabolism and nitrogen losses and improve outcome remains to be further investigated. Due to the study design of the included studies, our result should be verified by large, well-designed RCTs using NB-based strategy in the future.

## Data availability statement

The original contributions presented in the study are included in the article/Supplementary material, further inquiries can be directed to the corresponding author/s.

## Author contributions

Y-BZ contributed to the conception of the study, analysis, and drafting of the article. YY contributed to data collection and analysis. YX contributed to revisions of this manuscript. H-BH contributed to design and was responsible for the integrity of the work as a whole, from inception to publication of the article. All authors contributed to the article and approved the submitted version.

## Conflict of interest

The authors declare that the research was conducted in the absence of any commercial or financial relationships that could be construed as a potential conflict of interest.

## Publisher's note

All claims expressed in this article are solely those of the authors and do not necessarily represent those of their affiliated organizations, or those of the publisher, the editors and the reviewers. Any product that may be evaluated in this article, or claim that may be made by its manufacturer, is not guaranteed or endorsed by the publisher.

## Abbreviations

CI: confidence interval
CRRT: continuous renal replacement therapy
ICU: intensive care unit
LOS: length of stay
MD: mean difference
MV: mechanical ventilation
NB: nitrogen balance
NOS: Newcastle-Ottawa Scale
OR: odds ratio
RCTs: randomized controlled trials
SD: standard deviations.
