# Supplementary material for: Nitrogen balance and outcomes in critically ill patients: A systematic review and meta-analysis
Source: Front Nutr. 2022 Aug 22;9:961207. doi: 10.3389/fnut.2022.961207 (PMC9441883; doi:10.3389/fnut.2022.961207)

Nitrogen balance and Outcomes in Critically Ill Patients: A Systematic Review and Meta-Analysis

Yi-Bing Zhu[1], MD; Yan Yao[2], MD; Yuan Xu[2], MD; Hui-Bin Huang[2*] MD

***Corresponding author:**

Hui-Bin Huang, Email: hhba02922@btch.edu.cn.

Additional file

Additional file 1
Checklist following PRISMA guideline.....1
Additional file 2
Search strategy.....5
Additional file 3
Studies needed for full-reviewed but not included in the current meta-analysis.....7
Additional file 4
Summarizing finding of included studies for the hypophosphatemia and prognosis in ICU patients.....8
Additional file 5
Forest plot of comparison: change of nitrogen balance over the follow up.....9
Additional file 6
Forest plot of comparison: protein intake.....10
Additional file 7
Forest plot of comparison: calorie intake.....11
Additional file 8
Forest plot of comparison: length of stay in hospital.....12

Additional file 1

PRISMA 2009 checklist

Section/topic	#	Checklist item	Reported on page #
**TITLE**			
Title	1	Identify the report as a systematic review, meta-analysis, or both.	1
**ABSTRACT**			
Structured summary	2	Provide a structured summary including, as applicable: background; objectives; data sources; study eligibility criteria, participants, and interventions; study appraisal and synthesis methods; results; limitations; conclusions and implications of key findings; systematic review registration number.	2
**INTRODUCTION**			
Rationale	3	Describe the rationale for the review in the context of what is already known.	4
Objectives	4	Provide an explicit statement of questions being addressed with reference to participants, interventions, comparisons, outcomes, and study design (PICOS).	5
**METHODS**			
Protocol and registration	5	Indicate if a review protocol exists, if and where it can be accessed (e.g., Web address), and, if available, provide registration information including registration number.	6
Eligibility criteria	6	Specify study characteristics (e.g., PICOS, length of follow-up) and report characteristics (e.g., years considered, language, publication status) used as criteria for eligibility, giving rationale.	6
Information sources	7	Describe all information sources (e.g., databases with dates of coverage, contact with study authors to identify additional studies) in the search and date last searched.	6
Search	8	Present full electronic search strategy for at least one database, including any limits used, such that it could be repeated.	6 and Appendix file 2
Study selection	9	State the process for selecting studies (i.e., screening, eligibility, included in systematic review, and, if applicable, included in the meta-analysis).	6

Data collection process	10	Describe method of data extraction from reports (e.g., piloted forms, independently, in duplicate) and any processes for obtaining and confirming data from investigators.	7
Data items	11	List and define all variables for which data were sought (e.g., PICOS, funding sources) and any assumptions and simplifications made.	7
Risk of bias in individual studies	12	Describe methods used for assessing risk of bias of individual studies (including specification of whether this was done at the study or outcome level), and how this information is to be used in any data synthesis.	7
Summary measures	13	State the principal summary measures (e.g., risk ratio, difference in means).	7
Synthesis of results	14	Describe the methods of handling data and combining results of studies, if done, including measures of consistency (e.g., I^2) for each meta-analysis.	7-8
Risk of bias across studies	15	Specify any assessment of risk of bias that may affect the cumulative evidence (e.g., publication bias, selective reporting within studies).	7
Additional analyses	16	Describe methods of additional analyses (e.g., sensitivity or subgroup analyses, meta-regression), if done, indicating which were pre-specified.	8
**RESULTS**			
Study selection	17	Give numbers of studies screened, assessed for eligibility, and included in the review, with reasons for exclusions at each stage, ideally with a flow diagram.	9<br>Figure 1
Study characteristics	18	For each study, present characteristics for which data were extracted (e.g., study size, PICOS, follow-up period) and provide the citations.	9<br>Table 1
Risk of bias within studies	19	Present data on risk of bias of each study and, if available, any outcome level assessment (see item 12).	9
Results of individual studies	20	For all outcomes considered (benefits or harms), present, for each study: (a) simple summary data for each intervention group (b) effect estimates and confidence intervals, ideally with a forest plot.	9
Synthesis of results	21	Present results of each meta-analysis done, including confidence intervals and measures of consistency.	10-11
Risk of bias across studies	22	Present results of any assessment of risk of bias across studies (see Item 15).	Appendix 4
Additional analysis	23	Give results of additional analyses, if done (e.g., sensitivity or subgroup analyses, meta-regression [see Item 16]).	10-11

**DISCUSSION**			
Summary of evidence	24	Summarize the main findings including the strength of evidence for each main outcome; consider their relevance to key groups (e.g., healthcare providers, users, and policy makers).	12-16
Limitations	25	Discuss limitations at study and outcome level (e.g., risk of bias), and at review-level (e.g., incomplete retrieval of identified research, reporting bias).	16-17
Conclusions	26	Provide a general interpretation of the results in the context of other evidence, and implications for future research.	18
**FUNDING**			
Funding	27	Describe sources of funding for the systematic review and other support (e.g., supply of data); role of funders for the systematic review.	20

Additional file 2

Search Strategy : (Database: PubMed Embase Cochrane library ; Search completed 15th Jan 2022)

PubMed

(((("Critical Care"[Mesh]) OR ((((critical care[Title/Abstract]) OR (critically ill[Title/Abstract])) OR (intensive care[Title/Abstract])) OR ((((((((((((((Critical Illness[Title/Abstract]) OR (Critical Care[Title/Abstract])) OR (intensive care units[Title/Abstract])) OR (Burn units[Title/Abstract])) OR (coronary care units[Title/Abstract])) OR (respiration, artificial[Title/Abstract]))) OR (ventilators, mechanical[Title/Abstract])) OR (pulmonary ventilation[Title/Abstract])) OR (respiratory insufficiency[Title/Abstract])) OR (multiple organ failure[Title/Abstract])) OR (systemic inflammatory response syndrome[Title/Abstract])) OR (respiratory distress syndrome, adult[Title/Abstract])) OR (sepsis[Title/Abstract])) OR (shock, septic[Title/Abstract]))))) AND ((nitrogen balance) OR (nitrogen excretion))) AND (((((mortality) OR (death)) OR (survival)) OR (die)) OR (died))

Embase

No. Query

#21. #5 AND #17

#20. 'died':ab,ti AND [embase]/lim

#19. 'survival':ab,ti AND [embase]/lim

#18. death':ab,ti AND [embase]/lim

#17. #6 OR #7 OR #8 OR #9 OR #10 OR #11 OR #12 OR #13 OR #14 OR #15 OR #16

#16. 'critically ill':ab,ti AND [embase]/lim

#15. 'critical care':ab,ti AND [embase]/lim

#14. 'intensive care'/exp

#13. 'bacteremia':ab,ti AND [embase]/lim

#12. 'septic shock':ab,ti AND [embase]/lim

#11. 'sepsis':ab,ti AND [embase]/lim

#10. 'wound':ab,ti AND [embase]/lim

#9. trauma':ab,ti AND [embase]/lim

#8. 'icu':ab,ti AND [embase]/lim

#7. 'critical illness':ab,ti AND [embase]/lim

#6. 'intensive care':ab,ti AND [embase]/lim

#5. #1 OR #2 OR #3 OR #4

#4. 'netrogen excretion:ab,ti AND ([embase]/lim OR
[medline]/lim)
#3. 'netrogen excretion '/exp
#2. 'netrogen balance:ab,ti AND ([embase]/lim OR [medline]/lim)
#1. 'netrogen balance'/exp

Cochrane library

ID Search
#1 ("intensive care"):ti,ab,kw (Word variations have been searched)
#2 ("critically ill"):ti,ab,kw (Word variations have been searched)
#3 ("critical care"):ti,ab,kw (Word variations have been searched)
#4 ("critical illness"):ti,ab,kw (Word variations have been searched)
#5 ("Burn"):ti,ab,kw (Word variations have been searched)
#6 ("acute respiratory distress syndrom"):ti,ab,kw (Word variations have been searched)
#7 ("truma"):ti,ab,kw (Word variations have been searched)
#8 ("septic shock"):ti,ab,kw (Word variations have been searched)
#9 #1 OR #2 OR #3 OR #4 OR #5 OR #6 OR #7 OR #8
#10 ("netrogen balance"):ti,ab,kw(Word variations have been searched)
#11 ("netrogen excretion"):ti,ab,kw(Word variations have been searched)
#12 #10 AND #11
#13 #9 AND #12

Additional file 3

Table S3: Studies needed for full-reviewed but not included in the current meta-analysis (n=7 trials).

No	Study	Reason of exclusion
1	Allingstrup MJ, Esmailzadeh N, Wilkens Knudsen A, Espersen K, Hartvig Jensen T, Wiis J, Perner A, Kondrup J. Provision of protein and energy in relation to measured requirements in intensive care patients. Clin Nutr. 2012 Aug;31(4):462-8.	Reported without NB associated survival data
2	Arabi YM, Al-Dorzi HM, Mehta S, Tamim HM, Haddad SH, Jones G, McIntyre L, Solaiman O, Sakkijha MH, Sadat M, Afesh L, Kumar A, Bagshaw SM, Aldawood AS; PermiT Trial Group. Association of protein intake with the outcomes of critically ill patients: a post hoc analysis of the PermiT trial. Am J Clin Nutr. 2018 Nov 1;108(5):988-996.	Reported without NB associated survival data
3	J Dickerson RN, Pitts SL, Maish GO 3rd, Schroeppel TJ, Magnotti LJ, Croce MA, Minard G, Brown RO. A reappraisal of nitrogen requirements for patients with critical illness and trauma. J Trauma Acute Care Surg. 2012 Sep;73(3):549-57.	Reported without NB associated survival data
4	Balasubramanian S, Tran DH, Serra M, Parker EA, Diaz-Abad M, Deepak J, McCurdy MT, Verceles AC. Assessing calorie and protein recommendations for survivors of critical illness weaning from prolonged mechanical ventilation - can we find a proper balance? Clin Nutr ESPEN. 2021 Oct;45:449-453.	Reported without NB associated survival data
5	Dreydemy G, Coussy A, Lannou A, Petit L, Biais M, Carrié C. Augmented Renal Clearance, Muscle Catabolism and Urinary Nitrogen Loss: Implications for Nutritional Support in Critically Ill Trauma Patients. Nutrients. 2021 Oct 11;13(10):3554.	Reported without NB associated survival data
6	Rao M, Sharma M, Juneja R, Jacob S, Jacob CK. Calculated nitrogen balance in hemodialysis patients: influence of protein intake. Kidney Int. 2000 Jul;58(1):336-45.	Reported without ICU admission
7	van der Heijden A, Verbeek MJ, Schreurs VV, Akkermans LM, Vos A. Effect of increasing protein ingestion on the nitrogen balance of mechanically ventilated critically ill patients receiving total parenteral nutrition. Nutr Hosp. 1993 May-Jun;8(5):279-87.	Irrelevant to the current research
8	Danielis M, Lorenzoni G, Azzolina D, Iacobucci A, Trombini O, De Monte A, Gregori D, Beltrame F. Effect of Protein-Fortified Diet on Nitrogen Balance in Critically Ill Patients: Results from the OPINiB Trial. Nutrients. 2019 Apr 28;11(5):972.	Reported without NB associated survival data

Additional file 4

Table S6: Quality assessment and overall risk of bias of included studies

First author / year	Patient selection				Comparability	Outcome			Risk of bias
	Representation of the exposed cohort	Selection of the non-exposed cohort	Ascertainment of exposure	Outcome of interest not present at start	Comparability of cohorts on the basis of the design or analysis	Assessment of outcome	Was follow-up long enough for outcomes to occur	Adequacy of follow up of cohorts	
Berbel 2014	★	★	★	★	☆★	★	★	★	8
Bufarah 2018	★	★	★	★	☆★	★	★	★	8
Kim 2020	★	★	★	☆	★★	★	★	★	8
Kritmetapak 2016	★	★	★	★	★★	★	★	★	9
Ponce 2012	★	★	★	★	☆	★	★	★	8
Scheinkestel 2003	★	★	★	★	★★	★	★	★	9
Felicetti-Lordani 2017	★	★	★	★	★★	★	★	★	9
Buckley 2021	★	★	★	☆	★★	★	★	★	8

Abbreviations: H=high quality; M=moderate quality; L= low quality.

Note: A study was given a maximum of one point in each item within the patient selection and outcome domains and given a maximum of two points for the Comparability domain with the following criteria:

1. **Representation of the exposed cohort**: Studies received 1 point if they recruited consecutive series of adult patients with NB tested, or all included patients, or did not miss a large number of patients.
2. **Selection of the non-exposed cohort**: Studies received 1 point if both groups of patients that provided NB-associated survival information (defined by each author) were recruited from the same cohort.
3. **Ascertainment of exposure**: Studies received 1 point if they had been demonstrated abnormal NB levels.
4. **Outcome of interest was not present at the start of the study**: Studies received points if they demonstrated the outcome of interest was not present at the start of the study.
5. **Comparability:** Studies received points if they controlled the disease severity (i.e., SOFA, SAPS3, ISS, or APACHE II scores) (1 point); or any additional important factors such as age, gender or ethnicities, comorbidities, or there were no significant differences in initial NB levels between two groups (1 point).
6. **Assessment of outcome**: Studies received 1 point if they had independent blind assessment or record linkage.
7. **Was follow-up long enough for outcomes to occur**: Studies received 1 point if they followed up until at least either inpatient mortality or for 30 days or had adequate record linkage.

8. **Adequacy of follow-up for cohorts**: Studies received 1 point if all recruited subjects were followed up, or the number lost to follow-up was unlikely to introduce bias (≤10%.

Additional file 5

Forest plot of comparison: change of nitrogen balance over the follow up

Study or Subgroup	Survival Mean	Survival SD	Survival Total	Non-Survival Mean	Non-Survival SD	Non-Survival Total	Weight	Mean Difference IV, Fixed, 95% CI
Berbel 2014	4.7	7.4	98	0.2	10.1	35	1.6%	4.50 [0.85, 8.15]
Ponce 2012	7.15	1.2	86	3	1.3	44	98.4%	4.15 [3.69, 4.61]
**Total (95% CI)**			**184**			**79**	**100.0%**	**4.16 [3.70, 4.61]**

Heterogeneity: Chi² = 0.03, df = 1 (P = 0.85); I² = 0%

Test for overall effect: Z = 17.83 (P < 0.00001)

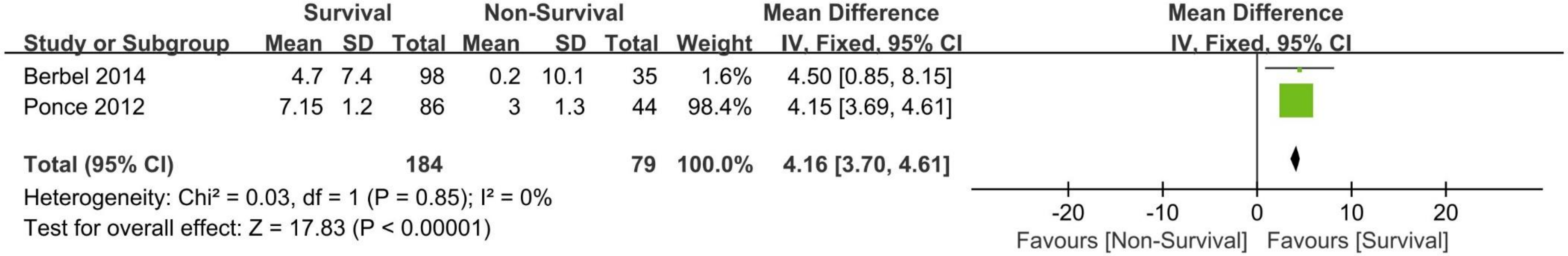

Additional file 6

Forest plot of comparison: protein intake

	Improved NB			Non-improved NB				Mean Difference
Study or Subgroup	Mean	SD	Total	Mean	SD	Total	Weight	IV, Fixed, 95% CI
Buckley 2021	1.2	0.4	5	0.8	0.8	24	23.3%	0.40 [-0.07, 0.87]
Kim 2020	1.94	0.63	39	1.28	0.54	38	76.7%	0.66 [0.40, 0.92]
**Total (95% CI)**			**44**			**62**	**100.0%**	**0.60 [0.37, 0.83]**

Heterogeneity: Chi² = 0.88, df = 1 (P = 0.35); $I^2 = 0\%$

Test for overall effect: Z = 5.12 (P < 0.00001)

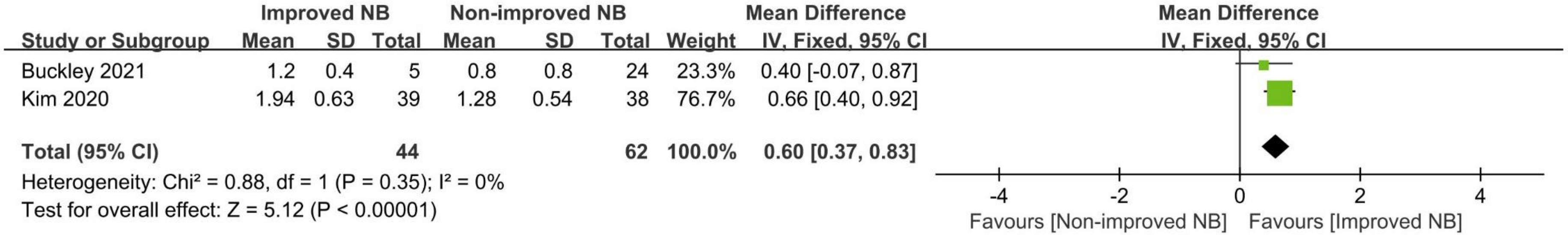

Additional file 7

Forest plot of comparison: calorie intake

Study or Subgroup	Improved NB Mean	Improved NB SD	Improved NB Total	Non-improved NB Mean	Non-improved NB SD	Non-improved NB Total	Weight	Mean Difference IV, Fixed, 95% CI
Buckley 2021	17	3	5	11	9	24	37.3%	6.00 [1.54, 10.46]
Kim 2020	25.3	7.5	39	21.5	7.9	38	62.7%	3.80 [0.36, 7.24]
**Total (95% CI)**			**44**			**62**	**100.0%**	**4.62 [1.90, 7.35]**

Heterogeneity: Chi² = 0.59, df = 1 (P = 0.44); I² = 0%

Test for overall effect: Z = 3.32 (P = 0.0009)

Mean Difference

IV, Fixed, 95% CI

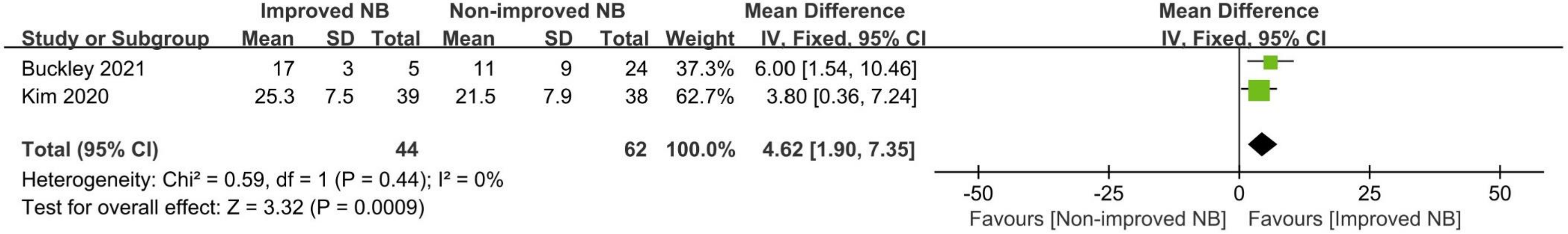

Additional file 8

Forest plot of comparison: length of stay in hospital

Study or Subgroup	Improved NB Mean	Improved NB SD	Improved NB Total	Non-improved NB Mean	Non-improved NB SD	Non-improved NB Total	Weight	Mean Difference IV, Fixed, 95% CI
Buckley 2021	38	43	5	20	13	24	14.3%	18.00 [-20.05, 56.05]
Kim 2020	38	37	39	33.5	32.6	38	85.7%	4.50 [-11.07, 20.07]
**Total (95% CI)**			**44**			**62**	**100.0%**	**6.44 [-7.97, 20.84]**

Heterogeneity: Chi² = 0.41, df = 1 (P = 0.52); I² = 0%

Test for overall effect: Z = 0.88 (P = 0.38)

Mean Difference IV, Fixed, 95% CI

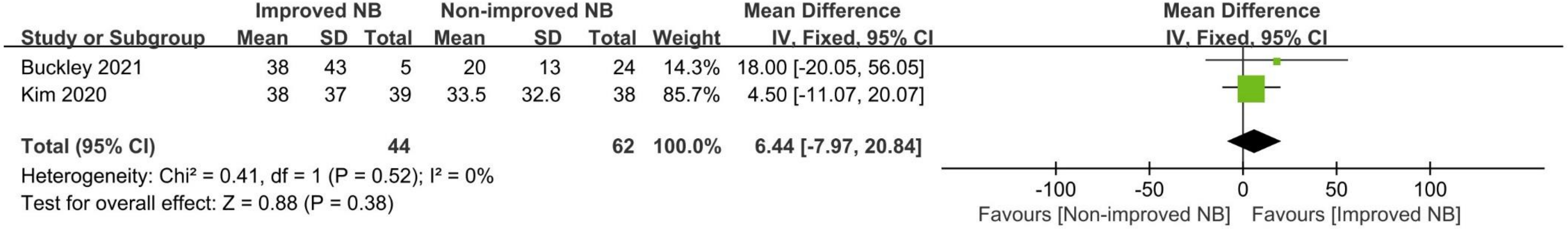

Supplement: Supplementary file 1 [file Data_Sheet_1.pdf]
